# Current status and potential challenges of mesenchymal stem cell-based therapy for malignant gliomas

**DOI:** 10.1186/s13287-018-0977-z

**Published:** 2018-08-24

**Authors:** Qing Zhang, Wei Xiang, Dong-ye Yi, Bing-zhou Xue, Wan-wan Wen, Ahmed Abdelmaksoud, Nan-xiang Xiong, Xiao-bing Jiang, Hong-yang Zhao, Peng Fu

**Affiliations:** 1 Department of Neurosurgery, Union Hospital, Tongji Medical College, Huazhong University of Science and Technology, Ave. Jiefang No.1277, Wuhan, 430022 People’s Republic of China; 2 Department of Cardiology, Beijing Anzhen Hospital, Capital Medical University, No. 2, Anzhen Road, Chaoyang District, Beijing, 100029 People’s Republic of China

**Keywords:** Mesenchymal stem cells (MSCs), Gliomas, MSC-based therapy, Current status, Potential challenges

## Abstract

Glioma, which accounts for more than 30% of primary central nervous system tumours, is characterised by symptoms such as headaches, epilepsy, and blurred vision. Glioblastoma multiforme is the most aggressive, malignant, and lethal brain tumour in adults. Even with progressive combination treatment with surgery, radiotherapy, and chemotherapy, the prognosis for glioma patients is still extremely poor. Compared with the poor outcome and slowly developing technologies for surgery and radiotherapy, the application of targeted chemotherapy with a new mechanism has become a research focus in this field.

Moreover, targeted therapy is promising for most solid tumours. The tumour-tropic ability of stem cells, including neural stem cells and mesenchymal stem cells, provides an alternative therapeutic approach. Thus, mesenchymal stem cell-based therapy is based on a tumour-selective capacity and has been thought to be an effective anti-tumour option over the past decades. An increasing number of basic studies on mesenchymal stem cell-based therapy for gliomas has yielded complex outcomes.

In this review, we summarise the biological characteristics of human mesenchymal stem cells, and the current status and potential challenges of mesenchymal stem cell-based therapy in patients with malignant gliomas.

## Background

Gliomas are the most common aggressive primary central nervous system tumours in adults [1], and they lead to a severe economic burden all over the world [2, 3]. Currently, the standard therapy for malignant gliomas is maximal safe surgical resection, postoperative radiotherapy, and then concomitant and adjuvant chemotherapy with temozolomide [4]. Due to the invasive growth features of malignant gliomas, complete resection is rarely achieved. Intra-operative fluorescence-guided technology, such as 5-aminolevulinic acid (5-ALA), could increase the resection proportion but does not improve the overall survival of patients with malignant gliomas [5]. Radiotherapy has been performed to treat patients with intra-cranial tumours since the 1950s [6], but the technology has been slow to develop, and one of the hallmarks of this disease is radiotherapy-resistant glioma stem cells (GSCs) [4]. Temozolomide is the most common and effective chemotherapy agent for patients with malignant gliomas, but the results depend on the promoter methylation status of O6-methylguanine methyltransferase (MGMT) [5]. However, the complex features of malignant glioma contribute to a poor prognosis. Glioblastoma (GBM), which has the worst prognosis, only has a 14.6-month median survival rate [7]. In recent years, with the increased understanding of the biological features of gliomas, an increasing number of precise targeted therapies have been generated.

Different types of stem and progenitor cells, including neural stem cells (NSCs), mesenchymal stem cells (MSCs), haematopoietic progenitor cells (HPCs), and embryonic stem cells (ESCs), have been shown to have the capacity for tumour-tropic homing and migration both in vitro and in vivo [8–11]. In tumour microenvironments, tumour cells and stromal cells can secrete several factors, including transforming growth factor (TGF)-β, stromal cell-derived factor-1α (SDF-1α), and vascular endothelial growth factor (VEGF), to recruit other cells into the tumour burden during tumour progression. Many reports have indicated that multipotent MSCs could migrate into tumour microenvironments in gliomas [12–14]. Based on the glioma tropism capacity, many basic and preclinical studies have used MSCs as cell vectors to deliver immune factors, anti-tumour proteins, anti-tumour microRNAs (miRNAs) or long non-coding RNAs (lncRNAs), suicide genes, and oncolytic viruses.

In this current review, we summarise the biological characteristics of human MSCs and present the principle of MSC-based therapy. Then, we analyse the current status and potential challenges in basic and preclinical studies for MSC-based therapy in patients with gliomas.

### Biological features of MSCs

**Fig. 1:**
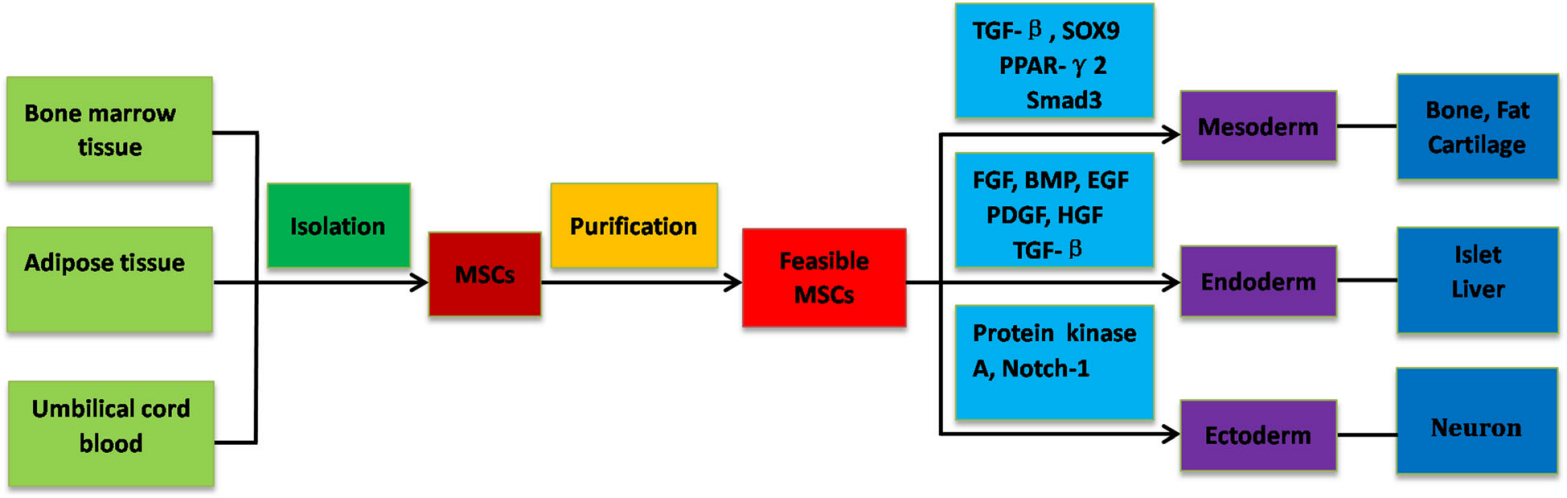
Source of mesenchymal stem cells (MSCs) and various signalling pathways regulating MSC differentiation. Multiple signalling pathways and cytokines have also been found to be involved in lineage commitment. BMP bone morphogenetic protein, EGF endothelial growth factor, FGF fibroblast growth factor, HGF hepatocyte growth factor, PDGF platelet-derived growth factor, TGF transforming growth factor

Friendenstein et al. reported the existence of MSCs in bone marrow in 1966 [15]. Subsequently, MSCs were widely and gradually found in many tissues [16–19]. MSCs were then gradually recognised as adult multipotent progenitor cells with the potential for self-renewal and differentiation into mesodermal and non-mesodermal lineages [20–22], but there was no definition marker for MSCs. In 2006, the International Society for Cellular Therapy (ISCT) defined the minimal criteria for MSCs according to their biological features: plastic adherent growth, positive expression of CD105, CD73, and CD90, negative expression of CD14, CD34, and CD45, and differentiation towards osteoblasts, adipocytes, and chondrocytes in vitro [23]. MSCs showed similar plastic adherent morphology from different sources [24], but they expressed slightly different cell surface markers, such as desmin, vascular endothelial (VE)-cadherin, α-smooth muscle actin (α-SMA), nestin, and nerval/glial antigen (NG2) (Table 1) [25]. MSCs from different sources could differentiate into multiple cell lines, such as bone [26], muscle [27], adipose [28], tendons [29], neurons [30], and myocardium [31], under specific in-vivo and in-vitro conditions. The differentiation pathway to a particular phenotype can be regulated by some gene events (Fig. 1).

**Table 1:** Cell surface markers of mesenchymal stem cells (MSCs) from four different sources

MSC source	Cell surface marker	References
Bone marrow	Positive: SH2, SH3, CD29, CD44, CD71, CD73, CD124, CD90, CD105, CD106, CD120a Negative: CD34, CD45, CD19, CD3, HLA-DR, CD31, CD11b	[12, 19, 24]
Adipose tissue	Positive: CD13, CD29, CD44, CD73, HLA-I, CD90, CD105, CD166, HLA-ABC, Negative: CD10, CD14, CD24, CD31, HLA-DR, CD36, CD38, CD45, CD49d, CD117, CD133, CD34, CD106, HLAII, SSEA4	[24, 44, 56, 72]
Umbilical cord	Positive: CK8, CK18, CK19, CD10, CD13, HLA-I, CD29, CD73, CD105, CD106, CD90, CD44, CD73 Negative: CD14, CD31, CD33, CD34, HLA-DR, CD45, CD38, CD79, CD133, VWF	[9, 16, 24]
Human glioma	Positive: CD90, CD105, CD73, CD44, CD151, α-SMA, desmin, VE-cadherin, NG2, STRO-1, HLA-I Negative: CD31, CD34, CD133, CD45, HLA-DR, CD14, CD19, Nestin, SMM	[25, 85–87]

The regulation of the biological features of MSCs is complex and multi-layered. The microenvironment conditions in different sources is thought to be the main reason for the slight differences in biological characteristics and genome patterns of MSCs. In vitro, TGF-β, bone morphogenetic proteins, insulin, dexamethasone, and other differentiation factors could induce MSCs into multiple cell lines [32–35]. Several signalling pathways, such as the TGF-β superfamily [36, 37], Hedgehog [38], and Wnt signalling [39], regulate and control the biological characteristics of MSCs. Recently, some new mechanisms, including miRNAs [40, 41] and lncRNAs [42], were reported to regulate the biological features. With a better understanding of MSCs, studies have aimed to modulate the biological characteristics of MSCs to treat several diseases, such as myocardial infarction, nerve injury, and arthritis.

### Tumour-specific tropism of MSCs

Recently, tumour-associated MSCs (TA-MSCs) have been regarded as integral components involved in several hallmarks of different tumours, such as initiation, promotion, progression, and metastasis. Some of these TA-MSCs already exist in many tissues, but some of them are recruited into tumour microenvironments to take part in the progression of tumours via the production of various growth factors, chemokines, and cytokines and to cross-talk with tumour cells [43, 44].

MSCs are rarely found in the brains of normal mice and humans, and these MSCs with classical features are located in a vascular niche [25, 43]. An increasing number of studies have reported that MSCs could be recruited into the tumour microenvironment of gliomas. For example, SP-DiI-labelled human MSCs, but not fibroblasts, were detected in U87 tumour masses after intra-vascular or intra-cranial administration in vivo [45]. However, the exact mechanism of the tumour-tropism of MSCs in a glioma microenvironment has not yet been fully elucidated. SDF-1α, VEGF, platelet-derived growth factor (PDGF), endothelial cell growth factor (EGF), TGF-β1, interleukin (IL)-8, and monocyte chemoattractant protein-1 (MCP-1) have been found to be secreted by glioma cells or stromal cells and to contribute to tumour-tropism of MSCs [12–14].

Based on their tumour-tropism and facile ability to cross the blood-brain barrier, MSCs have been utilised as carrying vectors for glioma therapy.

### MSC-based therapy for gliomas

MSC-based therapy for gliomas is a promising novel approach to transport different genes, proteins, and viruses. Various therapy approaches could suppress the growth of gliomas through different mechanisms, such as immunotherapy, suicide protein therapy, virus-based therapy, cytotoxic factor-based therapy, and anti-angiogenesis therapy, which are discussed more in the following sections.

#### Immunotherapy

Over the past decade, the ability of tumours to evade the immune system has always been a significant research direction for the rapid development of tumour immunotherapy. Malignant gliomas could escape the host immune system by secretion of immunosuppressive agents, inhibition of T-cell proliferation, and reduction in immune responses [46, 47]. Thus, the delivery of genes encoding cytokines, including interleukins (ILs) and interferon (IFN) family genes, to stimulate an immune response has been studied as an immunotherapy strategy in gliomas.

In 2004, Nakamura et al. found that intra-cranial administration of gene-modified MSCs expressing IL-2 could migrate towards a glioma site [48] and then infiltrate CD4 and CD8 lymphocytes, thus inducing strongly specific and curative anti-tumour immunity. Subsequently, researchers applied the same strategy using MSCs transduced to express other interleukins, such as IL-12, IL-7, and IL-18, and discovered that the anti-tumour effects of these agents were closely associated with enhancement of T-cell infiltration and tumour-specific T-cell responses, as well as the noting the occurrence of similar therapeutic efficacy in vivo [49–51]. In another attempt to target the immune system, Nakamizo et al. found that modified MSCs released soluble protein IFN-β, which significantly extended the survival of animals with established intra-cranial gliomas, and the inhibition of tumour cell growth correlated with dose-dependent increases [45].

**Fig. 2:**
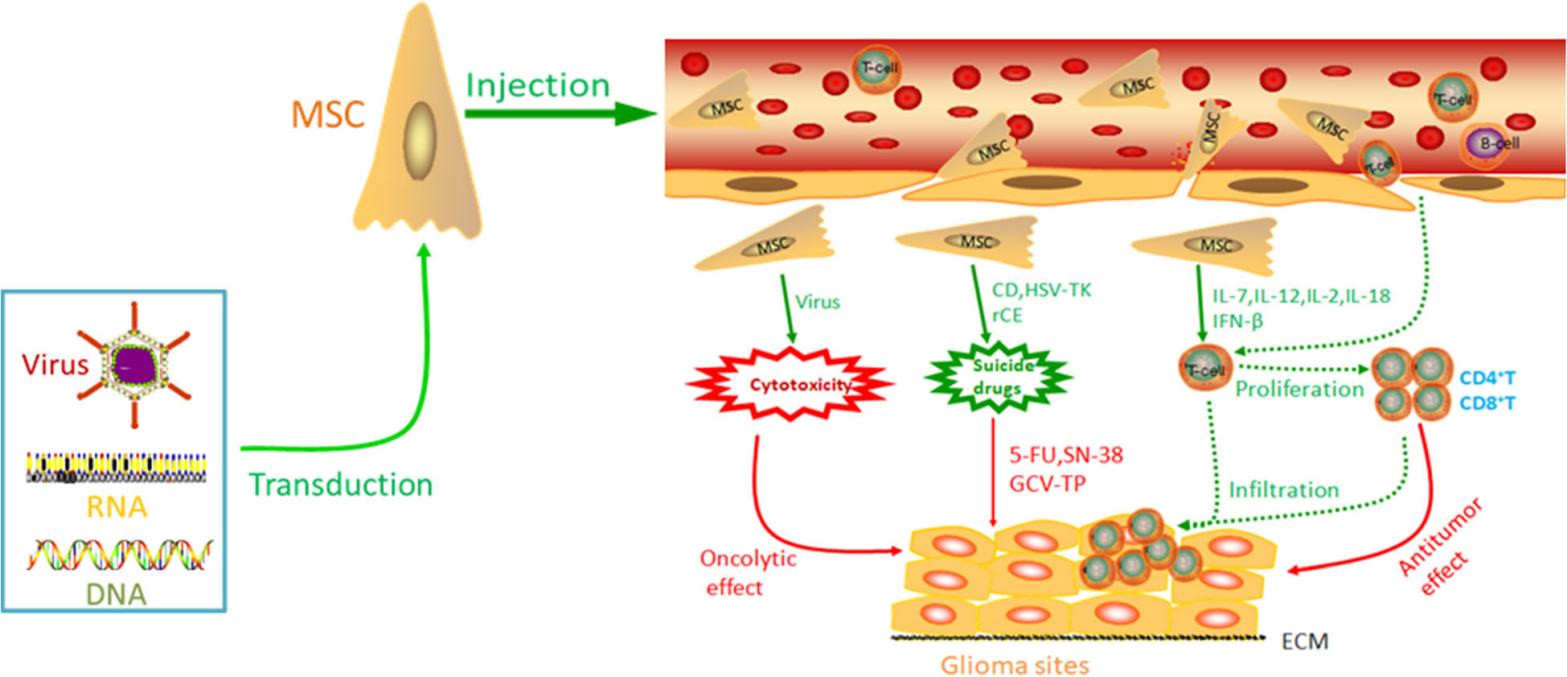
The pattern of mesenchymal stem cell (MSC)-based therapy studies for glioma. By means of tumour-specific tropism of MSCs, BMSCs, AT-MSCs, or UC-MSCs can be transduced to deliver anticancer agents such as TRAIL, interferon (IFN-β and IFN-γ) and interleukins (IL-2, IL-7, IL-18, and IL-12) directly to glioma sites to kill tumour cells or to regulate immune responses. MSCs can also be engineered with enzymes to convert pro-drugs into active drugs at the glioma site. For example, MSCs engineered to express yeast cytosine deaminase (CD), herpes simplex virus thymidine kinase (HSV-TK), and rabbit carboxylesterase (rCE) can convert systemically administered anti-tumour pro-drugs (5-fluorocytosine (5-FC), ganciclovir, and CPT-11, respectively) to their active form at the glioma site and thereby inhibit glioma growth while limiting peripheral toxicity. In addition, MSCs loaded with oncolytic adenovirus CRADs and Delta-24-RGD have been shown to have activity against glioma. 5-FU 5-fluorouracil, ECM extra-cellular membrane, SN-38 7-ethyl-10-hydroxycamptothecin, TP triphosphate

This review provides a summary of currently open and recruiting MSC-based immune-therapy studies for gliomas, as shown in Table 2.The patterns of MSC-based therapy studies for glioma are particularly illuminated in Fig. 2. The results showed that MSCs can be genetically engineered to express cytokines and augment the immune response via enhancing CD4^+^ and CD8^+^ T-cell infiltration and then stimulating subsequent cascade immune networks; these modified MSCs can be exploited to a therapeutic advantage against gliomas.

**Table 2:** Summary of currently open and recruiting mesenchymal stem cell (MSC)-based immunotherapy studies for glioma

MSC source (species)	Tumour type (species)	Route of administration	Experimental animal	Immunomodulatory gene	Year	References
Bone marrow (rat)	9 L (rat)	Intra-tumoural/contralateral	Fischer rat	IL-2	2004	[48]
Bone marrow (rat)	N32 (rat)	Intra-tumoural	Fischer rat	IL-7	2010	[50]
Bone marrow (human)	U87 (human)	Intra-tumoural/intra-carotid	Nude mice	IFN-β	2005	[45]
Bone marrow (rat)	C6 (rat)	Intra-tumoural	Spraguee-Dawley rat	IL-18	2009	[49]
Umbilical cord blood (human)	GL26 (mouse)	Contralateral/ipsilateral	C57BI/6 mice	IL-12	2011	[51]
Umbilical cord blood (human)	U87 (human)	Intra-tumoural/contralateral	Nude mice	TRAIL	2008	[71]

*IFN* interferon, *IL* interleukin, *TRAIL* tumour necrosis factor-related apoptosis-inducing ligand

#### Suicide protein-based therapy

Suicide protein-based therapy is a widely applied form of gene therapy in the cancer field. This approach entails mRNA encoding a pro-drug-activating enzyme (suicide protein) transduced into MSCs, the injection of these MSCs into the tumour sites, and the subsequent conversion of non-toxic pro-drugs into toxic pro-drugs, leading to regression of tumour cells in vivo [52, 53]. To date, the most commonly studied suicide genes in gliomas include herpes simplex virus thymidine kinase (HSV-TK) [54], cytosine deaminase/5-fluorocytosine (CD/5FC) [55], and rabbit carboxylesterase (rCE)/CPT-11 [56].

The HSV-TK/GCV system has been most reported in glioma treatment. This system is based on the ability of HSV-TK to efficiently phosphorylate the pro-drug ganciclovir to its monophosphate state, which is further phosphorylated by cellular enzymes to GCV-triphosphate (GCV-TP) [57]. MSCs expressing HSV-TK would be more feasible for clinical applications than the method using NSC therapy [54].

Later, De Melo et al. designed a strategy using adipose-derived MSCs (AT-MSCs) expressing HSV-TK combined with GCV, which was able to exert a cytotoxic effect on U87 cells in vitro and diminish tumour size [58, 59]. Similarly, data has shown that a TK-MSC combination with valproic acid could selectively exert a profound bystander effect on glioblastoma cells in vivo and that it did not injure normal brain tissues [60, 61]. This combined treatment significantly inhibited tumour growth and prolonged survival compared with glioma-bearing mice treated with MSC-TK in the absence of valproic acid (VPA) [58, 59].

Cytosine deaminase (CD) is another pro-drug-activating enzyme that can convert the non-toxic pro-drug 5-fluorocytosine (5-FC) to toxic 5-fluorouracil (5-FU), which effectively inhibits tumour growth. Early in 2012 a related study reported the use of CD-expressing MSCs combined with 5-FC for the treatment of intra-cranial rat gliomas and protected normal brain tissue from damage [62]. The CD/5-FC system demonstrated a potent bystander effect, with the ability to kill tumour cells even when the MSCs and tumour cells were not in direct contact, leading to the invading glioma cells becoming extensively disordered [63]. This system may represent a promising new therapeutic approach for highly invasive malignant gliomas. rCE enzymes can efficiently convert the pro-drug CPT-11 (irinotecan-7-ethyl-10-[4-(1-piperidino)-1-piperidino]carbonyloxycamptothecin) into the active drug SN-38 (7-ethyl-10-hydroxycamptothecin). Using the same enzyme/pro-drug therapy, Danks et al. explored intra-tumoural injection by combining genetically modified MSCs expressing rCE with CPT-11. The results showed that the therapy more effectively prolonged the survival of brain stem glioma-bearing rats than did treatment using only CPT-11 [64]. These strategies should provide an enhanced therapeutic effect for malignant gliomas.

#### Virus-based therapy

Oncolytic virotherapy is also a novel approach in which viruses are genetically modified to selectively replicate in tumour cells. The virus is released from its carrier at tumour sites and then selectively kills tumour cells and protects normal tissues from injury. However, the viral particles could be attacked and eliminated by the host immune system. In addition, the low-efficiency virus is far away from the tumour site. To overcome the barriers, tumour-tropic migratory cells may be used to deliver viral particles to the distant sites of tumours and shield a therapeutic virus from the host immune system [65].

Many studies using MSCs loaded with oncolytic adenovirus CRADs and Delta-24-RGD have demonstrated extended delivery of oncolytic viruses and prolonged survival of glioma-bearing animals treated with stem cell-mediated oncolytic virotherapy. In 2008, Sonabend et al. reported that MSCs provide CRAD delivery to distant glioma cells and that this delivery significantly enhanced the infection and apoptosis of tumour cells compared with injection of distant CRADs alone, showing a therapeutic advantage. Later, MSCs carrying Δ24-RGD (hMSC-Δ24) were injected into the carotid artery of mice and then migrated to tumour sites, resulting in inhibited growth and improved survival of mice. Taken together, previous results indicated that MSCs migrate and deliver CRADs and Delta-24-RGD to distant glioma cells and improved the outcome of oncolytic virotherapy for glioma [66–69]. These results have consistently shown that virus-loaded MSCs are capable of migrating towards glioma sites and releasing viral particles that selectively infect tumour cells, the effects of which ultimately kill tumour cells.

In the future, there is a possibility that tumour-specific antigens will replace the use of viruses in which the anti-viral immune response is caused by carrier proteins, and thus the virus could combine the benefits of virotherapy with immune-therapy to achieve the aim of treating cancer.

#### Cytotoxic factor-based therapy

One novel strategy of tumour treatment is to induce apoptosis of tumour cells. Tumour necrosis factor (TNF)-related apoptosis-inducing ligand (TRAIL) is a member of the tumour necrosis factor (TNF) superfamily and could induce apoptosis of tumour cells through activation of the TNF/CD95L axis and spare the majority of non-malignant cells [70]. Studies have shown that MSCs expressing TRAIL could migrate towards a glioma, maintain their stem-like properties, induce the cytotoxic effects of glioma cells, and show prolonged survival in glioma animal models. TRAIL-secreting MSCs have the migration capacity towards tumour cells and can directly target glioma. In 2008, Kim et al. used umbilical cord-derived MSCs (UC-MSCs) expressing TRAIL to demonstrate a reduction in tumour volume and improvement in survival rate in glioma-bearing mice in vivo compared with controls [71]. Subsequently, Choi et al. reported the therapeutic efficacy and safety of TRAIL-producing human AT-MSCs against glioma, providing significant data for clinical trials using MSCs with therapeutic genes against brain gliomas [72]. The above studies demonstrate that MSCs expressing cytotoxic factors could migrate to tumour sites and induce apoptosis of tumour cells. Therefore, these results suggest that MSCs expressing TRAIL could provide an interesting approach for anti-glioma therapy.

#### Anti-angiogenesis therapy

Angiogenesis is an important characteristic event in the development and progression of gliomas. The use of factors that inhibit angiogenesis is thought to be one of the strategies for glioma treatment.

Pigment epithelial-derived factor (PEDF) is a 50-kDa secreted glycoprotein that can activate the Fas/FasL pathway to induce endothelial cell death and regulate the balance between inducers and inhibitors of angiogenesis [73, 74]. Previously, Zhang et al. discovered that PEDF played an important role in angiogenesis and tumourigenesis of gliomas [75]. In 2013, Wang et al. proved that MSCs expressing PEDF effectively induced tumour cell apoptosis and inhibited angiogenesis, thereby decreasing tumour volume and prolonging the survival of glioma-bearing mice [74]. However, the molecular mechanism by which PEDF causes glioma apoptosis and anti-angiogenesis was not fully understood. Afterwards, Guo et al. found that the conditioned medium from phosphatase and tensin homologue (PTEN) mRNA-engineered MSCs induced U251 cell death via PI3K-AKT-mTOR pathways in vitro [76].

Thus, engineered MSCs act as an inhibitory molecular vehicle to promote apoptosis and attenuate angiogenesis in gliomas, and they may have the potential as a therapeutic agent in the clinical application of stem cell therapy against gliomas.

### Potential challenges

Although MSC-based therapy for malignant gliomas has become increasingly popular, all MSC-based therapies, similar to other stem cell-based therapies, appear to be limited in their effectiveness. Some recent studies on cross-talk between MSCs and glioma cells reveal the potential challenges to MSC-based therapy for gliomas.

#### Do MSCs support or suppress glioma growth?

There is no clear conclusion on whether MSCs themselves support or suppress the progression of glioma. AT-MSCs can increase the size of glioma tissue by a reduction in apoptosis and secretion of VEGF [77]. UC-MSCs can inhibit migration by the upregulation of PTEN and induce apoptosis of glioma cells by the downregulation of X-linked inhibitor of apoptosis (XIAP) [78]. Bone marrow-derived MSCs (BMSCs) can enhance the invasion of U373 but not U87 by overexpression of proteases [79]. AT-MSCs can promote the epithelial-mesenchymal transition in glioma cells [80]. Moreover, Schichor et al. found that the fusion of U87 and BMSCs facilitated the proliferation and migration of both in vitro, and the cross-talk of tumour cells and MSCs maintained the structural formation of syncytium [81]. Subsequently, Sun et al. showed that the fused cells gave rise to an enhanced angiogenesis of gliomas in vitro and in vivo and a stabilised vascular framework, implying an improvement in glioma growth [82].

Therefore, MSCs from different sources showed different abilities to promote or suppress the growth of glioma cells under different conditions. If these MSCs were investigated as delivery vectors, the effects of MSCs supporting or suppressing the growth of gliomas should also be considered.

#### Glioma-associated MSCs (gbMSCs)

gbMSCs were first isolated from fresh glioma tissues and identified by our team in 2014. We found that gbMSCs showed classical features of morphology, surface markers, and differentiation. The gbMSCs secrete different factors dependent on the inter-cellular cross-talk and hypoxia conditions [83]. Moreover, the percentage of gbMSCs in tumour samples correlated with the outcomes of patients with high-grade gliomas, whereas patients with a high percentage of gbMSCs had a poor overall survival rate [84]. Svensson et al. reported there were two distinct subpopulations of gbMSCs with different CD90 expression. CD90^−^ gbMSCs produced more VEGF and prostaglandin E2 (PGE2) than did CD90^+^ gbMSCs, and these two subpopulations had 211 differentially expressed genes but showed no mRNA differences [85]. Our team also found that CD90^−^ gbMSCs could differentiate into pericytes and contribute to neovascularization in the glioma microenvironment [25]. CD90^+^ gbMSCs could increase the growth and invasion of glioma cells under a serum deprivation condition (data not shown). Figueroa et al. also found that gbMSCs could increase the tumourigenicity of glioma stem cells by microRNA-1587 exosome transfer [86] and maintain the stemness of glioma stem cells by secretion of IL-6 [87]. All these data suggest the promoting role of gbMSCs in the aggression and progression of gliomas.

#### Malignant transformation of MSCs

As well as an assistant role of MSCs in tumour microenvironments, some studies have reported that MSCs can directly transform to malignant cells and have revealed a new challenge to MSC-based therapy for cancer. In 2004, Houghton et al. suggested that BMSCs were recruited to the sites of pre-cancerous lesions by inflammatory cytokines and then transformed into gastric cancer cells to promote cancer progression through a series of transformations [88]. Serakinci et al. reported that human adult MSCs had the neoplastic potential to contribute to mesenchymal tumour development after transducing the human telomerase reverse transcriptase (TERT) gene [89]. Recently, Tan et al. demonstrated that the proliferation and migration rate of the transformed mesenchymal stem cells (TMCs) were significantly increased compared with that of the MSCs in vitro and that TMCs led to tumourigenesis in an animal model [90]. These studies strongly suggest that MSC malignancy is possible under specific conditions, and there is a clear conclusion about the phenomenon and mechanism of malignant transformation of MSCs.

#### MSC-mediated immunosuppression

In tumour microenvironments, MSCs could support tumour growth by suppression of host immune responses. Once MSCs are recruited into a tumour microenvironment, they can secrete several ligands to help tumour cells build the immunosuppressive environment by recruiting monocytes, macrophages, and myeloid-derived suppressor cells. These immune cells then inhibit the anti-tumour immune responses of T cells [43]. In gliomas, some researchers found that CD90^−^ gbMSCs showed stronger ability for tumour immunosuppression than their CD90^+^ counterparts [85]. However, there are still too few publications about the suppressive role of MSCs in glioma.

## Conclusions

Currently, MSC-based therapy for gliomas has become an increasing focus of research. MSCs are regarded as effective vectors to deliver therapeutic agents to tumour sites. Despite MSC-related studies making great strides in the field of glioma treatment, there remain some powerful challenges. Moreover, there are no on-going clinical trials on MSC-based therapy for malignant gliomas. In conclusion, MSC-based therapy for glioma is still in its infancy, and we need a better understanding of the biological consequences of MSC-based therapy before it is widely used in the treatment of patients with malignant gliomas.

## Abbreviations

5-FC: 5-Fluorocytosine
AT-MSC: Adipose-derived mesenchymal stem cell
BMSC: Bone marrow-derived mesenchymal stem cell
CD: Cytosine deaminase
EGF: Endothelial cell growth factor
ESC: Embryonic stem cell
gbMSC: Glioma-associated mesenchymal stem cell
GSC: Glioma stem cell
HPC: Haematopoietic progenitor cell
HSV-TK: Herpes simplex virus thymidine kinase
IFN: Interferon
IL: Interleukin
MCP-1: Monocyte chemoattractant protein-1
MSC: Mesenchymal stem cell
NSC: Neural stem cell
PDGF: Platelet-derived growth factor
PEDF: Pigment epithelial-derived factor
PGE2: Prostaglandin E2
PTEN: Phosphatase and tensin homologue
rCE: Rabbit carboxylesterase
SDF-1α: Stromal cell-derived factor-1α
TA-MSC: Tumour-associated mesenchymal stem cell
TGF: Transforming growth factor
TMC: Transformed mesenchymal stem cell
TNF: Tumour necrosis factor
TRAIL: Tumour necrosis factor-related apoptosis-inducing ligand
UC-MSC: Umbilical cord-derived mesenchymal stem cell
VEGF: Vascular endothelial growth factor
XIAP: X-linked inhibitor of apoptosis
